# Two new species of *Octomeria* (Orchidaceae, Pleurothallidinae) and two new country records from Ecuador

**DOI:** 10.3897/phytokeys.274.177794

**Published:** 2026-04-29

**Authors:** Leisberth Vélez-Abarca, Gabriel A. Iturralde, Henry X. Garzón-Suárez, Diego Gutiérrez del Pozo, J. R. Kuethe, Luis E. Baquero, Marco M. Jiménez

**Affiliations:** 1 Grupo Científico Calaway Dodson: Investigación y Conservación de Orquídeas del Ecuador, Quito, 170510, Pichincha, Ecuador Facultad de Ingeniería y Ciencias Aplicadas, Universidad de Las Américas, UDLA Quito Ecuador; 2 Herbario HUTPL, Departamento de Ciencias Biológicas, Universidad Técnica Particular de Loja, San Cayetano Alto s/n 11-01-608, Loja, Ecuador Faculty of Sciences, University of Auckland Auckland New Zealand; 3 Grupo de Investigación en Biodiversidad, Medio Ambiente y Salud BIOMAS, Carrera de Ingeniería Agroindustrial, Facultad de Ingeniería y Ciencias Aplicadas, Universidad de Las Américas, UDLA, Vía a Nayón, Quito 170124, Ecuador Departamento de Ciencias Biológicas, Universidad Técnica Particular de Loja Loja Ecuador; 4 Universidad Estatal Amazónica UEA, Herbario Amazónico del Ecuador ECUAMZ, Carretera Tena a Puyo Km. 44, Carlos Julio Arosemena Tola 150950, Napo, Ecuador Grupo Científico Calaway Dodson: Investigación y Conservación de Orquídeas del Ecuador Quito Ecuador; 5 School of Environment, Faculty of Sciences, University of Auckland, City Campus for Science and Engineering, Symonds Street, Auckland, New Zealand Universidad Estatal Amazónica UEA, Herbario Amazónico del Ecuador ECUAMZ Carlos Julio Arosemena Tola Ecuador

**Keywords:** Morona Santiago, northern Andes, premontane forest, taxonomy, Zamora Chinchipe

## Abstract

*Octomeria* (Orchidaceae) is a genus of predominantly epiphytic herbs distributed from Belize to Argentina. Ecuador hosts 30 species of *Octomeria*, making it one of the most species-rich countries for the genus. In Ecuador, most species occur in the south-eastern province of Zamora-Chinchipe. Two new species of *Octomeria* (*O. ivouquillasii* and *O. tricarinata*) from Ecuador are described and illustrated. Taxonomic affinities between the new taxa and their nearest related species are discussed and information regarding habitat ecology is provided. Additionally, two related species (*O. oncidioides* and *O. odontoglossoides*) have been reported in south-eastern Ecuador for the first time. Additional notes regarding *O. candidae* are included, correcting the collection number of the type material.

## Introduction

The genus *Octomeria* R.Br. (Orchidaceae) comprises approximately 160 species distributed from Belize to northern Argentina, though the majority of species are found from the Guianas down to southern Brazil (Forster 2007; Forster et al. 2012; Karremans et al. 2019). Thirty species have been reported for Ecuador, where most notably 21 species are found in the Amazonian province of Zamora-Chinchipe alone (POWO 2025; Vélez-Abarca unpublished data). Many of these southern species were discovered during recent expeditions that led to the description of *Octomeria candidae* Vélez-Abarca, M.M Jiménez & Baquero, *O. jimenezii* Vélez-Abarca, *O. pacii* Vélez-Abarca, M.M Jiménez & Baquero and *O. panguiensis* Vélez-Abarca, M.M Jiménez & Baquero. These findings show the need for continued targeted expeditions within these sub-Amazonian regions of eastern Ecuador as there is a great potential to increase the known species for the province and the country (Vélez-Abarca et al. 2020, 2021, 2024).

Species of the genus are generally epiphytic (though rarely lithophytic) and range from caespitose to creeping in habit. Ramicauls may be short or long, with either sessile or petiolate leaves that orientate flat to conduplicate or even semi-terete to terete. Inflorescences consist of an abbreviated main axis from which single-flowered co-florescences are produced either successively or simultaneously in a fascicle at the apex of the ramicaul; these individual flowers are borne on short pseudopeduncles (Rojas-Alvarado and Karremans 2024). Sepals and petals are generally similar in shape, though usually their size is slightly unequal in relation. The lateral sepals are usually free, but can also be connate at the base. The lip may be entire or lobed, the column semi-terete with generally a subapical anther, a ventral stigma and the pollinarium composed of eight pollinia (except in *O. splendida* Garay & Dunst., which has six) (Forster et al. 2012).

From a phylogenetic perspective, recent molecular analyses of the subtribe Pleurothallidinae have confirmed that *Octomeria* constitutes a well-supported monophyletic lineage, provided that certain peripheral groups, such as the *O. parvifolia* alliance, are excluded (Karremans 2016). The genus is recognised as a member of the early diverging ‘*Octomeria* affinity’, occupying a basal position relative to more derived clades within the subtribe (Zhang et al. 2023). This early divergence in the evolutionary history of the Pleurothallidinae is consistent with the genus’s extensive biogeographical footprint; *Octomeria* exhibits one of the most widespread distributions in the Neotropics, ranging from Belize and the Antilles to northern Argentina and southern Brazil (Forster 2007; Luer 2010; POWO 2025). This vast range suggests a high degree of evolutionary plasticity and an early colonisation of diverse habitats, from the Amazonian lowlands to the montane forests of the Andes, where the genus continues to show a high rate of endemism and taxonomic novelty (Forster et al. 2012; Karremans et al. 2019).

In the course of preparing a taxonomic revision of the genus *Octomeria* for the southern Ecuadorian Amazon (Vélez-Abarca, unpublished data), two undescribed species, along with two new records for the country, were discovered. In this paper, the two species are described and illustrated, providing comparisons with morphologically similar species within the genus and offering information regarding their habitat and ecology. In addition, new records for both *O. odontoglossoides* Luer and *O. oncidioides* Luer have been added to the Ecuadorian flora and notes regarding their distribution and habitat have been provided.

## Material and methods

Descriptions and illustrations of the new species were prepared, based on fresh material collected in the southern Ecuadorian Amazon, under research permits granted by the Ministerio del Ambiente de Ecuador. Photographs of flowering individuals were taken using a Panasonic FZ300 camera with a Raynox DCR-250 50 mm and a Canon EOS 1100D camera, equipped with a 10 Kernel Pro Optics 58 mm macro and an EFS 18–55 mm lens. The composite plates were prepared using Adobe Photoshop CC 2021, applying digital calibration for all diagnostic structures through ImageJ (Schneider et al. 2012). Fresh flowers were preserved in a solution of 70% ethanol and 1% glycerol to accompany the holotypes. Herbarium specimens, photographs and original manuscripts for all morphologically similar species were examined using the specialised literature (Cogniaux 1896; Luer 2010; Vélez-Abarca et al. 2021). Holotypes of the newly-described species were deposited at the Universidad Técnica Particular de Loja Herbarium (HUTPL). The extent of occurrence (EOO) and area of occupancy (AOO) were calculated for each species using the GeoCAT tool (https://geocat.iucnredlist.org/) (Bachman et al. 2011), set using the IUCN-recommended default cell width of 2 km. A preliminary conservation status was proposed for each new species following the criteria and categories outlined in the IUCN Red List guidelines (IUCN 2024).

## Taxonomic treatment

### Octomeria ivouquillasii

Taxon classification

Plantae

AsparagalesOrchidaceae

Vélez-Abarca & M.M.Jiménez sp. nov.

3DFDB67E-C20E-5842-9AAC-2D25146E8AF8

urn:lsid:ipni.org:names:77379350-1

**Figure 1.**
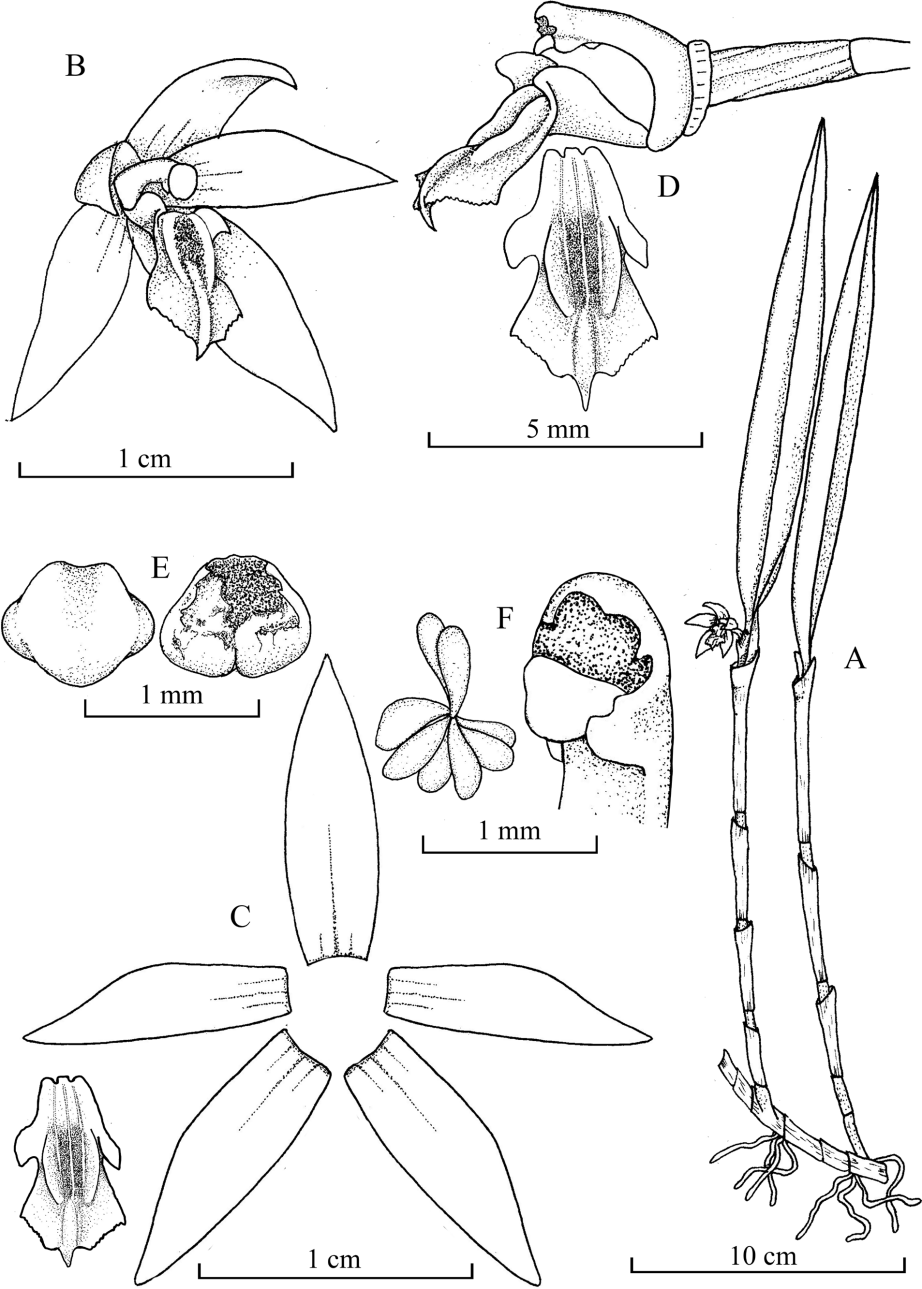
*Octomeria ivouquillasii* Vélez-Abarca & M.M.Jiménez. **A**. Habit; **B**. Flower in ¾ view; **C**. Dissected perianth; **D**. Ovary, column and lip in lateral view and lip adaxial view; **E**. Anther cap, adaxial (left) and abaxial (right) views; **F**. column apex and pollinia. Illustration made by L. Vélez-Abarca, based on the holotype.

**Figure 2.**
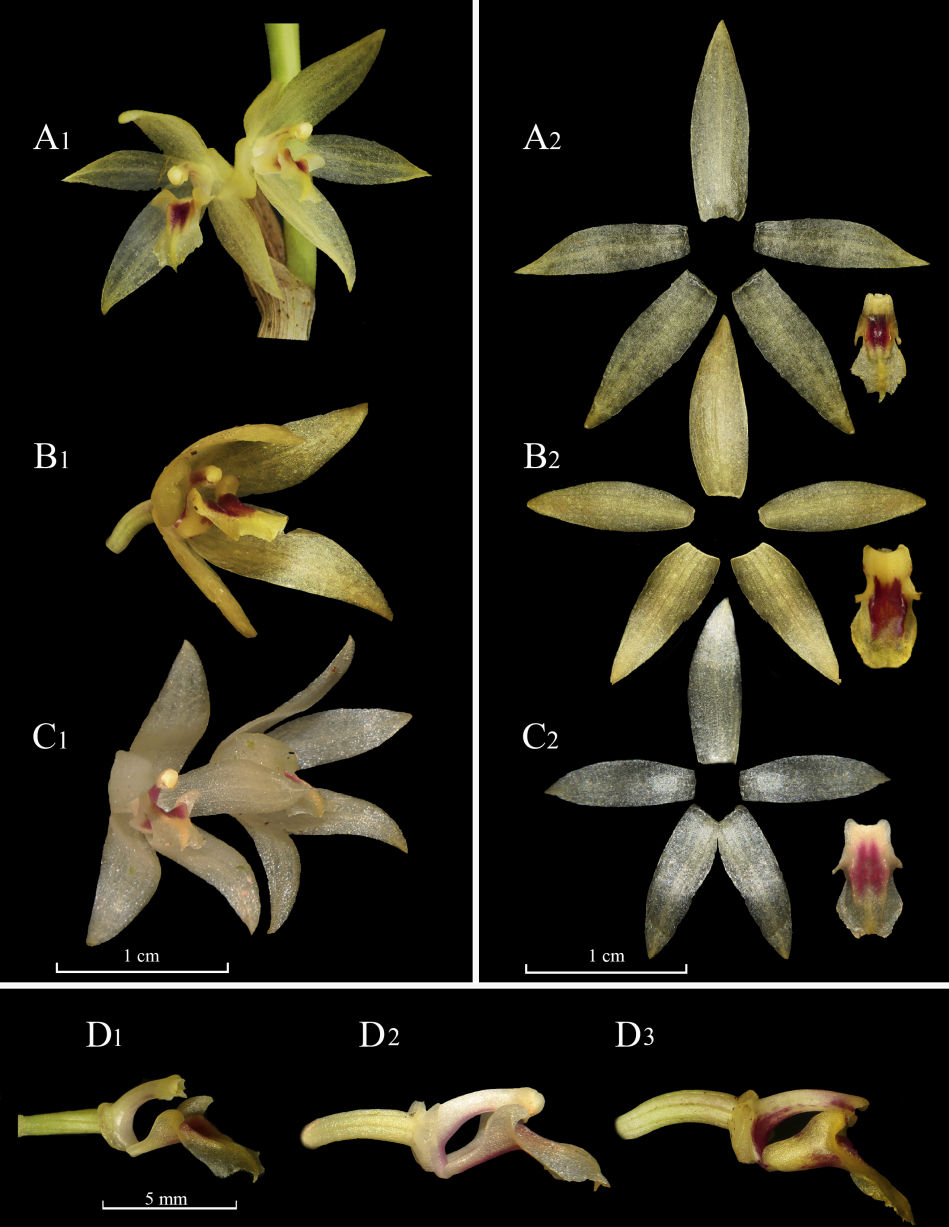
Comparative pictures amongst *Octomeria ivouquillasii*, *O. seegeriana* and *O. robusta* (from top to bottom). **A1, A2, D1**. Flower and dissected perianth of *O. ivouquillasii*; **B1, B2, D3**. Flower and dissected perianth of *O. seegeriana*; **C1, C2, D2**. Flower and dissected perianth of *O. robusta*; **D1–D3**. Lateral view of the column, lip and ovary, of the respective species. Photographs by Marco M. Jiménez (*O. ivouquillasii* and *O. seegeriana*) and L. Vélez-Abarca (*O. robusta*). Plate elaborated by L. Vélez-Abarca and H.X. Garzón.

Figs 1, 2: A1, B1, C, D; Table 1

#### Diagnosis.

*Octomeria ivouquillasii* is similar to the robust, repent members of the *O. grandiflora* complex (including *O. seegeriana* Kraenzl. (Fig. 2: A2, B2) in its large plant size and creeping habit, but differs by having slender, compressed ramicauls (vs. terete), a reduced pseudopeduncle 1.0–1.2 mm long (vs. 2–3 mm long), an obconic pedicel (vs. cylindrical) and a rhomboid apical lobe of the lip with acute, strongly apiculate apex and a central, longitudinal pseudo-callus extending over the apical third (vs. ovate, apex broadly truncate to retuse, sometimes minutely apiculate, without a central callus).

**Table 1.** Morphological differences amongst *Octomeria ivouquillasii*, *O. seegeriana* (syn. *O. grandiflora*) and *O. robusta* (syn. *O. grandiflora*). Descriptions taken from: (1) this study; (2, 3) Luer (2010), Ulloa Ulloa et al. (2018).

Species	*O. ivouquillasii* (1)	*O. seegeriana* (2)	*O. robusta* (3)
**Plant**	Up to 40 cm tall, repent rhizome, stout, 4 mm thick, 0.5–1.5 cm long between ramicauls	Up to 33 cm tall, repent, rhizome, stout, 4–5 mm thick, 1–2 cm long between ramicauls	Up to 23 cm tall, ascending to shortly repent or caespitose, rhizome stout, 0.5–1.0 cm long between ramicauls
**Ramicaul**	Slightly compressed, 10–20 cm long	Terete, 5–15 cm long	Terete, 6–22 cm long
**Leaf**	Narrowly lanceolate, acute, 12–15 × 1.4–1.7 cm, petiole 1.8 mm long.	Elliptical, acute, 8–15 × 1.5–2.5 cm, petiole 0.5–1.5 mm long	Elliptical, acute, 7–16 × 1.0–2.5 cm, petiole 1.0–1.5 mm long
**Sepals**	3-veined. Dorsal sepal elliptic-ovate, subacute to acuminate, 10–12 × 2–3 mm; lateral sepals elliptic, acuminate, 10–12 × 2–3 mm	5-veined. Dorsal sepal elliptic-ovate, acute, 9–14 × 5 mm; lateral sepals elliptic, slightly oblique, acute, 9–14 × 5 mm	4-to 5-veined Dorsal sepal elliptic-ovate, acute, 11–12 × 4–5 mm, 5–7-veined; lateral sepals elliptic-ovate, slightly oblique, acute, 10–11 × 3.5–5.0 mm.
**Petals**	Narrowly ovate, acuminate, 10–12 × 2–3 mm, 3-veined	Ovate, acute, 9–14 × 3.0–4.5 mm, 5-veined	Narrowly ovate, acute, 9–10 × 3.0–4.5 mm, 3–5-veined
**Lip**	Trilobed, 4.5–5.0 × 2.0–2.3 mm expanded, the apical lobe rhomboid, acute apex, retuse with a pseudo-callus extending in the central region below the apical third	Trilobed, 5–8 × 3–4 mm expanded, the apical lobe broadly truncate to retuse, without a callus	Trilobed, 7–8 × 4.0–4.5 mm expanded, the apical lobe erose, ovate, apex broadly truncate to retuse, without a callus
**Lateral lobes of the lip**	Rounded, elongated, antrorse	Oblique, broadly uncinate, subacute, antrorse	Low, obtusely angled on the anterior margin

#### Type.

Ecuador • Zamora-Chinchipe: Cordillera del Cóndor, cerca de Tundayme, 3°36'35.83"S, 78°29'16.84"W, 1400 m elev., 23 May 2021, *M. Jiménez 1183* (holotype: HUTPL 15796!).

#### Description.

***Plant*** up to 40 cm tall, epiphytic, repent rhizome, stout, 4.0 mm thick, 0.5–1.5 cm long between ramicauls. Roots ca. 1.0 mm in diameter, flexuous, white. ***Ramicauls*** erect, 10–20 cm long, slightly compressed, with 3–5 internodes enclosed by tubular bracts. ***Leaf*** erect, coriaceous, narrowly lanceolate, acuminate, 12.0–15.0 × 1.4–1.7 cm, adaxially sulcate and abaxially carinate, margin entire, cuneate at the base on a petiole ca.1.8 mm long. ***Inflorescence*** a single-flowered co-florescence, produced successively from an abbreviated axis at the apex of the ramicaul; pseudopeduncle cylindrical, 1.0–1.2 mm long; flower bract 1, infundibuliform, membranaceous, 2.5–3.0 mm long; pedicel obconic, 2.5 mm long; ovary 2.5–3.8 mm long, cylindrical, sulcate, almost always twisted. Sepals and petals white-yellowish, translucent, glabrous, 3-veined, 10–12 × 2.0–3.0 mm. ***Dorsal sepal*** elliptic-ovate, subacute to acuminate. ***Lateral sepals*** elliptic, acuminate. ***Petals*** narrowly ovate, acuminate, 10–12 × 2–3 mm, 3-veined. ***Lip*** yellow, pigmented red purple on the first half of the disc, between the calli, oblong, three-lobed, glabrous, 4.5–5.0 × 2.0–2.3 mm, with lateral lobes antrorse, erect, rounded, elongated, the apical lobe rhomboid, acute, strongly apiculate, the disc broadly channelled between a pair of oblique yellow calli descending from the lateral lobes, a yellow pseudo-callus extending longitudinally below the apical third; base truncate, articulated to the foot of the column. ***Column*** semi-terete, falcate, 3.0–3.5 mm long, foot of the column 1.5 mm long, stigma ventral, clinandrium entire, margin slightly dentate. ***Anther cap*** white, subapical. ***Pollinia*** eight, in two series of four, yellow.

#### Distribution and habitat.

*Octomeria ivouquillasii* is so far known only from its type locality found along the western side of the Cordillera del Cóndor in the Zamora-Chinchipe Province, Ecuador (Fig. 7). The species was found growing at an elevation of 1400 m, corresponding with low premontane forests on the foothills of a sandstone plateau.

#### Etymology.

The specific epithet honors Ivo Alberto Uquillas Bermeo, Ecuadorian artist (painter and sculptor) from Manabí Province, Ecuador. This species is dedicated to his contributions to the visual arts and his numerous impactful cultural works of monumental sculpture and public art across Ecuador.

#### Conservation status.

*Octomeria ivouquillasii* is known from a single locality; therefore, the Extent of Occurrence (EOO) cannot be calculated and the Area of Occupancy (AOO) is the minimum possible value of 4 km^2^ following IUCN (2024) guidelines. The only known population occurs within an area surrounded by mining concessions (Fig. 7) and lies outside any protected area. Severe deforestation and habitat loss associated with large-scale mining have been documented in the region (Mestanza-Ramón et al. 2022; Mena-Quintana et al. 2025). Although insufficient sampling suggests the species may occur elsewhere, access to surrounding areas is increasingly restricted due to the presence of mining companies, which often limit entry and restrict biological research.

Given the limited available information, the species could be categorised as Data Deficient (DD). However, considering its extremely restricted AOO and the clear, ongoing threats to its habitat, we recommend listing it as Critically Endangered [CR B2ab(iii)] (IUCN 2024) under a precautionary approach.

#### Taxonomic notes.

The original description of *Octomeria grandiflora* (Fig. 4: D1) by Lindley (1842) defined the species with a mid-lobe of the lip as “*obovato-cuneatâ denticulatâ fissâ*”, describing a widened apex with denticulate margins and a cleft or divided centre (*fissâ*). This morphology differs from *O. ivouquillasii*, which presents a strictly rhomboid mid-lobe with entire margins and an apex that, rather than being divided, projects into an acute angle ending in a solid and prominent apex.

Luer (2010) maintained Lindley’s interpretation by illustrating *Octomeria grandiflora* with a broadly truncate to retuse apex. However, the new entity possesses a creeping habit and slightly compressed ramicauls that distance it from the typically cespitose forms with terete stems treated by Luer for *O. grandiflora*. For this reason, the diagnostic comparison is established with *O. seegeriana* (Fig. 2: A2, B2) and *O. robusta* (Fig. 2: A3, B3), taxa that have been currently synonymised under *O. grandiflora* (Ulloa Ulloa et al. 2018).

When contrasting the new entity with *Octomeria seegeriana*, both share the large plant size and its creeping habit. However, the new species is distinguished by possessing a reduced venation of only three veins in all floral segments (vs. five veins in sepals and petals). The new species is readily distinguishable by the smaller size of the lip 4.5–5.0 × 2.0–2.3 mm (vs. 5.0–6.0 × 3.0 mm); with an apical lobe rhomboid, acute, strongly apiculate apex; and a central longitudinal pseudo-callus projecting from the middle towards the apex (vs. ovate, apex broadly truncate to retuse, sometimes minutely apiculate) (Table 1).

Regarding *Octomeria robusta*, the structural divergence is even more pronounced due to the density of the venation, consistently presents a reduced venation of only 3 veins in both petals and sepals (vs. 5–7 veins in the sepals). Both share the large plant size and its creeping habit. The new species is readily distinguishable by the smaller size of the lip 4.5–5.0 × 2.0–2.3 mm (much larger lip, 7.0–8.0 × 4.0 mm); with apical lobe rhomboid, acute, strongly apiculate apex; and a central longitudinal pseudo-callus projecting from the middle towards the apex (vs. ovate, apex broadly truncate to retuse, sometimes minutely apiculate and without a central callus). The combination of reduced venation, the rhomboid-acute architecture of the lip and the presence of the central pseudocallus confirms the new entity as an independent species (Table 1).

### Octomeria tricarinata

Taxon classification

Plantae

AsparagalesOrchidaceae

Vélez-Abarca & M.M.Jiménez sp. nov.

0ACCD5CC-4004-521A-807A-908A29CDCF97

urn:lsid:ipni.org:names:77379351-1

**Figure 3.**
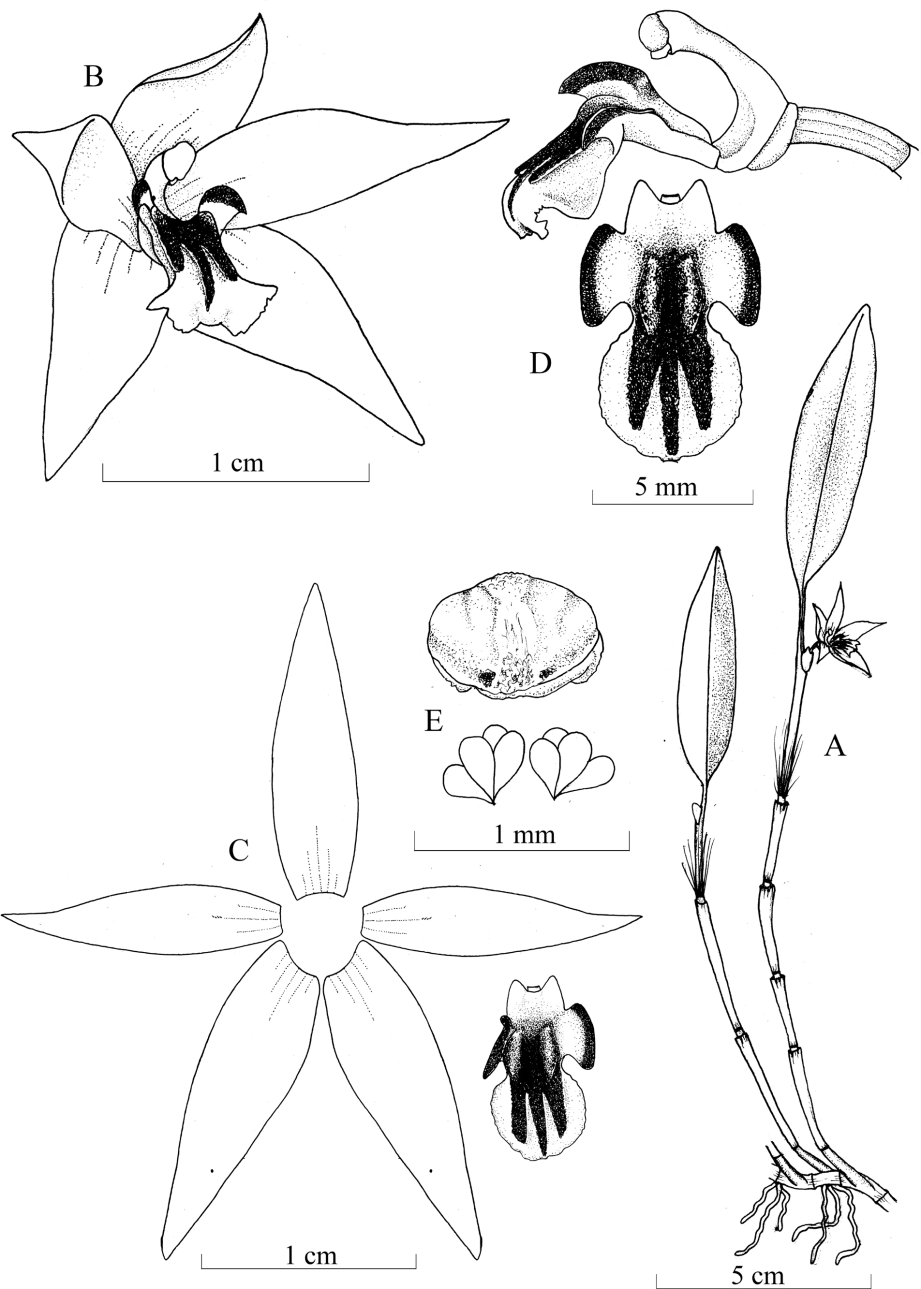
*Octomeria tricarinata* Vélez-Abarca & M.M.Jiménez. **A**. Habit; **B**. Flower in ¾ view; **C**. Dissected perianth; **D**. Ovary, column and lip in lateral view and lip adaxial view; **E**. Anther cap and pollinia. Illustration made by L. Vélez-Abarca, based on the type specimen.

**Figure 4.**
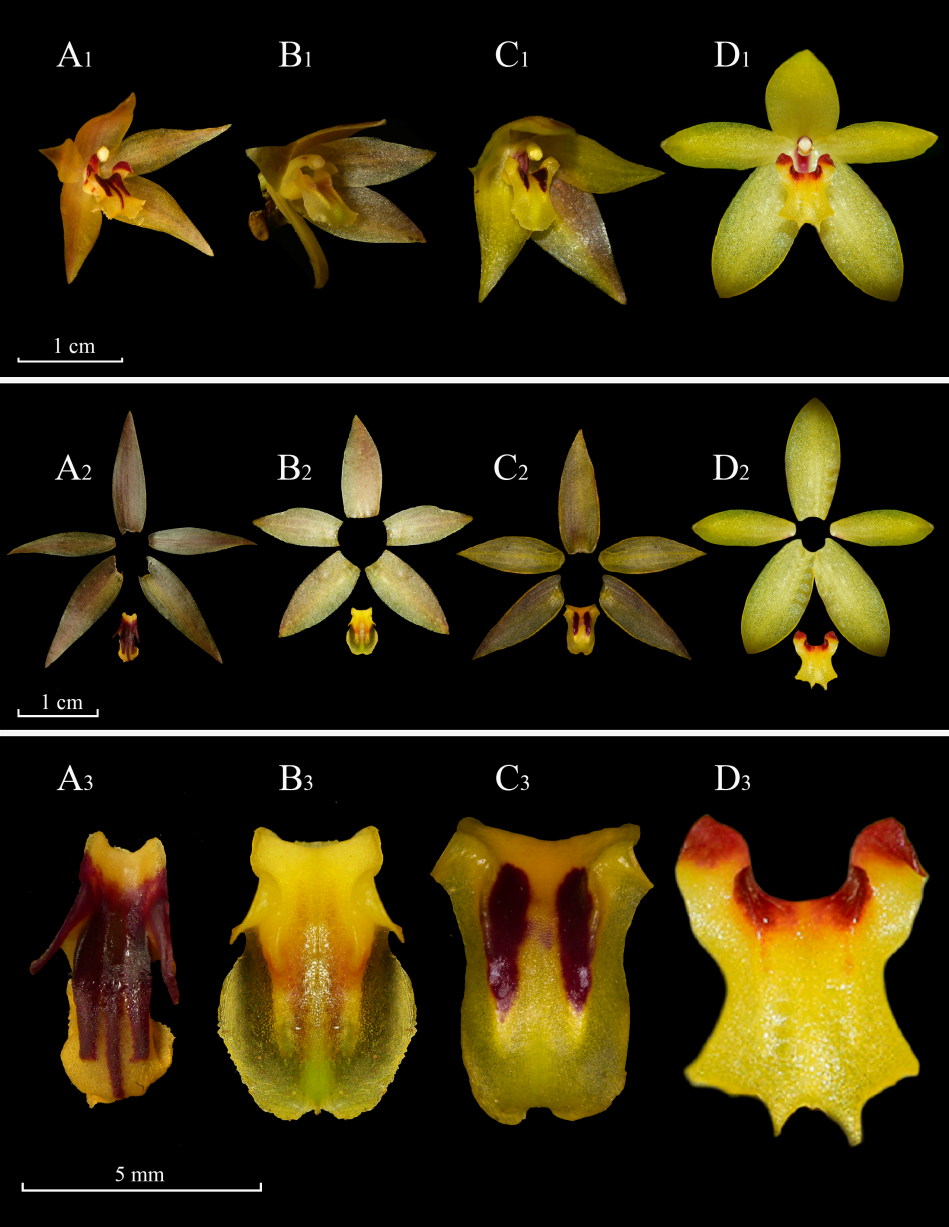
Morphological comparison amongst *Octomeria tricarinata*, *O. hirtzii*, *O. peruviana* and *O. grandiflora*. **A1–A3**. Flower, dissected perianth and adaxial view of the lip of *O. tricarinata*; **B1–B3**. Flower, dissected perianth and adaxial view of the lip of *O. hirtzii*; **C1–C3**. Flower, dissected perianth and adaxial view of the lip of *O. peruviana*; **D1–D3**. Flower, dissected perianth and adaxial view of the lip of *O. grandiflora*. Photographs by M. M. Jiménez (*O. tricarinata* and *O. hirtzii*), L. Ocupa-Horna (*O. peruviana*) and M. S. Rosim (*O. grandiflora*). Plate prepared by L. Vélez-Abarca and H.X. Garzón.

Figs 3, 4A–C; Table 2

#### Diagnosis.

*Octomeria tricarinata* is similar to *O. grandiflora* in the size of the plant and flower but differs by its shortly repent habit (vs. densely caespitose), shorter, linear-oblong leaves, 5.0–10.0 × 1.0–2.5 cm (vs. ovate to narrowly ovate, 8.0–20.0 × 1.0–2.0 cm), thinner lip 5.2–5.8 × 1.7–2.2 mm (vs. 6.0–9.0 × 4.0–6.0 mm), with three well-marked, dark red-purple calli (vs. a pair of yellow calli) and the apical lobe suborbicular, rounded (vs. subquadrate, broadly truncate to retuse).

**Table 2.** Morphological differences amongst *Octomeria tricarinata*, *O. hirtzii*, *O. peruviana* and *O. grandiflora*. Descriptions taken from: (1) this study; (2, 3, 4) Luer (2010).

Species	*O. tricarinata* (1)	*O. hirtzii* (2)	*O. grandiflora* (3)	*O. peruviana* (4)
**Plant**	Up to 30 cm tall, shortly repent, robust rhizome, 0.5–1.5 cm between ramicauls	Up to 60 cm tall, caespitose, without a thickening rhizome	Up to 20 cm or more tall, densely caespitose, without a thickened rhizome	Up to 70 cm tall, ascending to caespitose, rhizome thick
**Ramicaul**	Slender, laterally compressed, 11–25 cm long	Stout, terete, 30–42 cm long	Slender, terete, 5–20 cm long	Stout, laterally compressed, 30–45 cm long
**Leaf**	Linear-oblong, acute, 5–10 × 1.0–2.5 cm	Narrowly linear-oblong, acute,19–32 × 1.0–1.5 cm	More or less narrowly lanceolate, 8–20 × 1–2 cm	Narrowly ovate, sharply acute, 20–30 × 2.5–4.0 cm
**Sepals**	Dorsal sepal elliptical-ovate, acute to acuminate, 14.0–16.0 × 3.5–4.0 mm, 5-veined; lateral sepals elliptical-ovate, acute, 14.0–17.5 × 3.5–4.0 mm, 5-veined	Dorsal sepal elliptical, acute, 13 × 4.5 mm, 5-veined; lateral sepals elliptical, slightly oblique, acute, 13 × 4.5 mm, 5-veined	Dorsal sepal ovate-lanceolate, 10–18 × 3–5 mm, 5- to 7-veined; lateral sepals similar, fused at base, acute, 10–18 × 3–5 mm, 5–6-veined	Dorsal sepal elliptical, acute, 10–15 × 4–5 mm, 5-veined; lateral sepals, elliptical, slightly oblique, acute, 10–15 × 4–5 mm, 5-veined

#### Type.

Ecuador • Zamora-Chinchipe: Cerca de Tundayme, Cordillera del Cóndor, 3°34'53.32"S, 78°29'10.02"W, 886 m elev., 23 May 2021, *M. Jiménez 948* (holotype: HUTPL14720!).

#### Description.

***Plant*** up to 30 cm tall, epiphytic, shortly repent, robust rhizome 3.5 mm in diameter, 0.5–1.5 cm between ramicauls, thin, flexuous roots, yellowish-white. ***Ramicauls*** erect, slender, laterally compressed, 11.0–25.0 cm long, enclosed by three to five tubular sheaths. ***Leaf*** erect, coriaceous, linear-oblong, acute, 5.0–10.0 × 1.0–2.5 cm, petiole 1.0–1.3 cm long. ***Inflorescence*** a single-flowered co-florescence, produced successively in a fascicle from an abbreviated axis at the apex of the ramicaul; pseudopeduncles ca. 1 mm long; floral bracts 2 mm long; pedicel 2 mm long; ovary 4 mm long. ***Flowers*** yellow suffused with brown. ***Sepals*** free, elliptical-ovate, glabrous, acute, 5-veined; dorsal sepal 14.0–16.0 × 3.5–4.0 mm; lateral sepals 14.0–17.5 × 3.5–4.0 mm. ***Petals*** narrowly ovate, acute to acuminate, 13.0–13.2 × 3.1–3.2 mm, 3-veined. ***Lip*** yellow, oblong-trilobed, glabrous, 5.2–5.8 × 1.7–2.2 mm, apical lobe suborbicular, rounded, reflexed, with minutely erose margins, the lateral lobes yellow at the base and dark red-purple at the edge, erect, antrorse, rounded at the tip, axe-shaped, three dark red-purple, elevated, longitudinal calli emerging from the middle third, the central callus extending to the apex, the base truncate, articulated to the foot of a short column; column yellow, stained with red-purple, semi-terete, clavate, falcate, 5 mm long, clinandrium entire, margins irregular. ***Anther cap*** white, apical, the stigma ventral. ***Pollinia*** eight, yellow, obovate.

#### Distribution and habitat.

*Octomeria tricarinata* is currently endemic to Ecuador, though its distribution fringes the Peruvian border, making it plausible that it can be found within the northern Amazonian regions of this country. Within Ecuador, it is restricted to the eastern sub-Andean region of Morona-Santiago and Zamora-Chinchipe Provinces, where it is found at elevations ranging from 900 to 1200 m (Fig. 7). The type population was collected near Tundayme, along the foothills of the sandstone plateaus of the Cordillera del Cóndor. Forest here is dominated by species of *Sciodaphyllum* P.Browne, *Cyathea* Sm. and *Clusia* L. A smaller population was seen in San Juan Bosco at 1200 m elev. and a record from the Kalaglás River Valley (Luer 2010) at 1500 m elev. in Morona-Santiago appears to be present in a remnant of primary forest surrounded by grasslands.

#### Etymology.

This species is named in reference to the tricarinate callus of the mid-lobe of the lip, a diagnostic feature of this species.

#### Conservation status.

*Octomeria tricarinata* is currently known from three localities (here defined as geographically distinct areas in which a single mining or deforestation event may severely affect most or all individuals of the species) in south-eastern Ecuador, where it is found across a calculated extent of occurrence (EOO) of 100.8 km^2^ and an area of occupancy (AOO) of 12 km^2^. This species faces significant threats of habitat destruction at all known sites. El Pangui-Tundayme population occurs within an active mining concession where habitat degradation is ongoing. The San Juan Bosco and Kalaglás populations are located within fragmented forest remnants subject to further land transition, for example, selective logging or agricultural activities. Given the limited number of known individuals, the small AOO and the presence of direct threats to the habitat for all known localities, the species meets the criteria for Endangered (EN) under IUCN (2024) criterion B2ab(iii), with an AOO < 500 m^2^ and continuing decline forecast in the quality of habitat (Mestanza-Ramón et al. 2022; Mena-Quintana et al. 2025; Uvidia et al. 2025), potentially leading to Critically Endangered in the near future. Further field surveys are needed to determine whether additional populations exist and to continue monitoring the status of the currently known sites.

#### Additional material examined.

Ecuador • Morona-Santiago: Epiphytic in rainforest along Río Calagras, -3.271727, -78.527439, ca. 1500 m elev., collected 19 Sept 1980, flowered in cultivation 20 Oct 1981, *C. Luer 6534* (SEL-051389!); • San Juan Bosco, 3°05'02.4"S, 78°30'22.3"W, 1201 m elev., 26 Feb 2023, *H. Garzón-Suárez 185* (HUTPL 14797!).

#### Taxonomic notes.

The new species is most similar to *Octomeria grandiflora* (Fig. 4), but can be readily identified by having three dark red-purple calli present on the mid-lobe of the lip, along with other features mentioned in the diagnosis. In the short original description of *O. grandiflora*, Lindley (1842) explicitly mentioned it as the largest *Octomeria* known to “him”, with long ovate to narrowly ovate leaves making it different from the shorter linear-oblong leaves seen in *O. tricarinata*. Lindley also described the lip as having two fleshy lamellae on the disc, further contrasting the species with the three calli emerging from the middle third, as seen in *O. tricarinata*.

Other diagnostic features of the new species contrasting with *O. grandiflora* [as treated by Cogniaux (1896)] are the shortly repent habit (vs. densely caespitose); the rhizome being 3.5 mm in diameter with ramicauls up to 25 cm tall (vs. rhizomes about 4 mm in diameter, ramicauls 6–20 cm tall); linear-oblong leaves making them smaller than the ramicauls (vs. leaves more or less narrowly lanceolate and about the same length as the ramicaul), the elliptical-ovate sepals that are 14–16 mm long, erect (vs. the sepals ovate-lanceolate, 10–12 mm long), the lip with dolabriform lateral lobes, the midlobe rounded at the apex and the disc consisting of three calli emerging from the middle third (vs. the lip having oblong lateral lobes, the mid-lobe with a toothed apical margin and the callus consisting of two fleshy lamellae). *O. tricarinata* is also similar to *O. peruviana* and *O. hirtzii* from which it differs by similar traits as mentioned above (Table 2).

In his systematic treatment of *Octomeria*, Luer (2010: fig. 20b) illustrated a specimen from Morona-Santiago (Luer 6534, SEL) under the name *O. grandiflora*. However, our examination reveals that this specific individual possesses morphological characteristics such as the presence of three longitudinal calli on the mid-lobe that deviate from the original description of *O. grandiflora* (Lindley 1842) and the type of material from Brazil. These features align instead with the taxon described here as *O. tricarinata*. By clarifying these morphological distinctions, we clarify the circumscription of *O. grandiflora* and provide evidence for the recognition of *O. tricarinata* (Fig. 2) as a distinct entity.

##### Two new records of *Octomeria* from Ecuador

### Octomeria odontoglossoides

Taxon classification

Plantae

AsparagalesOrchidaceae

Luer, Monogr. Syst. Bot. Missouri Bot. Gard. 120: 102 (fig. 37). 2010.

10658570-420D-5C79-8851-A453B36F8CFC

#### Type.

Colombia • Without collection data. Purchased from a local collector, flowered in cultivation at Colomborquídeas, El Retiro, 20 Nov 1981, *C. Luer 6729* (Holotype: SEL-002480!).

#### Notes.

**Figure 5.**
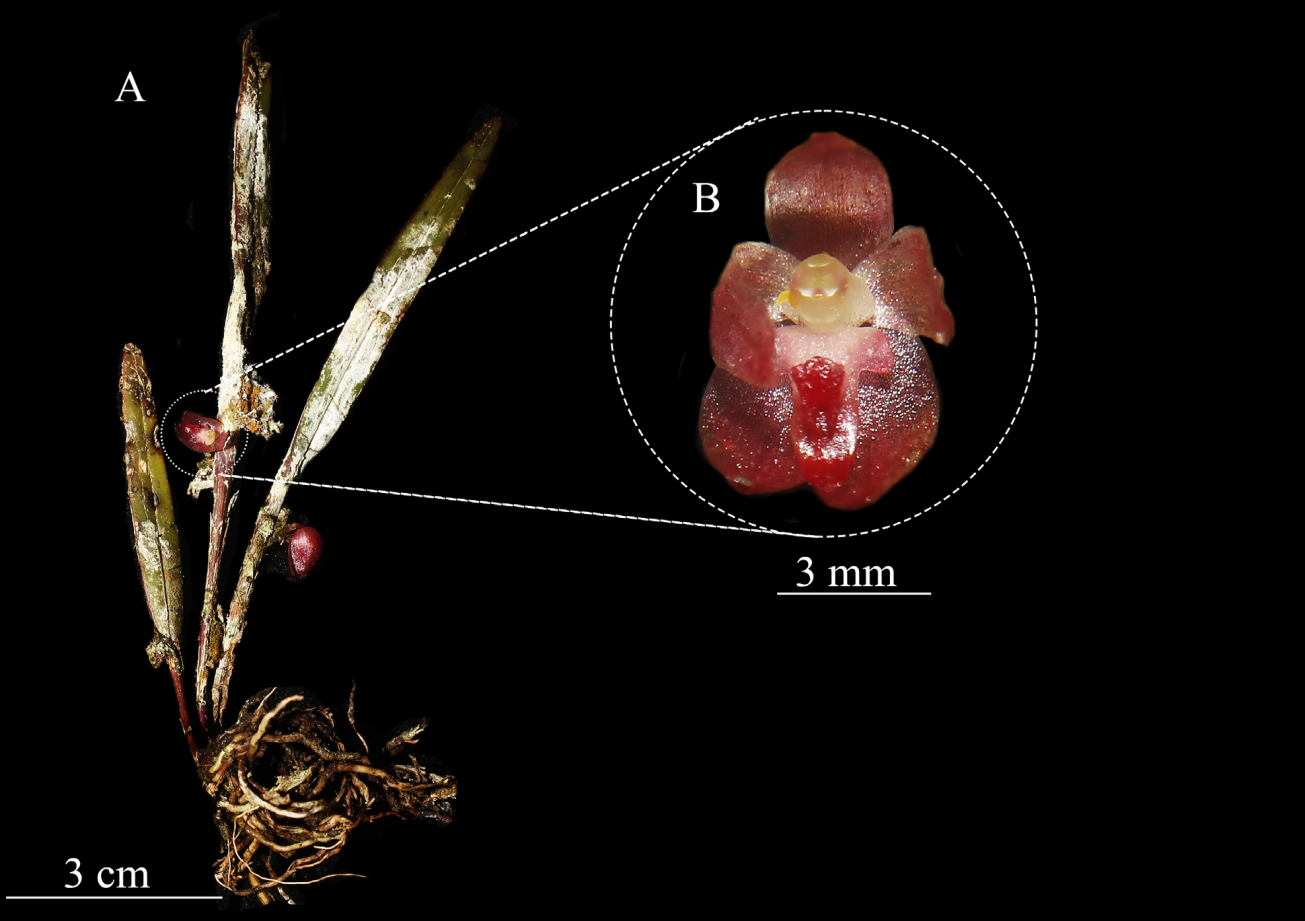
*Octomeria odontoglossoides*. **A**. Habit; **B**. Flower close-up. Plate elaborated by L. Vélez-Abarca, based on *L. Vélez LV-77*.

*Octomeria odontoglossoides* (Fig. 5) was previously known only from Colombia, an assessment based solely on the type specimen, which was collected without any location data. In 2023, this species was found in Zamora-Chinchipe, southern Ecuador, where it was seen near Tundayme in the El Pangui Canton. This represents the first record for Ecuador, although it is plausible that, given the lack of location data on the type material, the location of “Colombia” may have been allocated incorrectly. This species is found growing on tree trunks within premontane foothill forests between 900 and 1100 m in elevation of the Cordillera del Cóndor.

#### Specimen examined.

ECUADOR • Zamora-Chinchipe: El Pangui, cerca de Tundayme, 3°35'5.95"S, 78°29'36.06"W, 942 m elev., 18 Feb 2023, *L. Vélez-Abarca 77* (ECUAMZ 12215!).

### Octomeria oncidioides

Taxon classification

Plantae

AsparagalesOrchidaceae

Luer, Revista Soc. Boliv. Bot. 4(1): 11. 2003.

C1B34E9E-261B-5618-A94B-F8FAF9226A30

#### Type.

Bolivia • La Paz: Larecaja, Alto Llipi, above Santa Barbara, above Tipuani, 1200 m elev., collected by *A. Hirtz, Juan del Hierro & W. Teague*, 30 Aug 1991, *C. Luer 15383* (Holotype: MO).

#### Notes.

**Figure 6.**
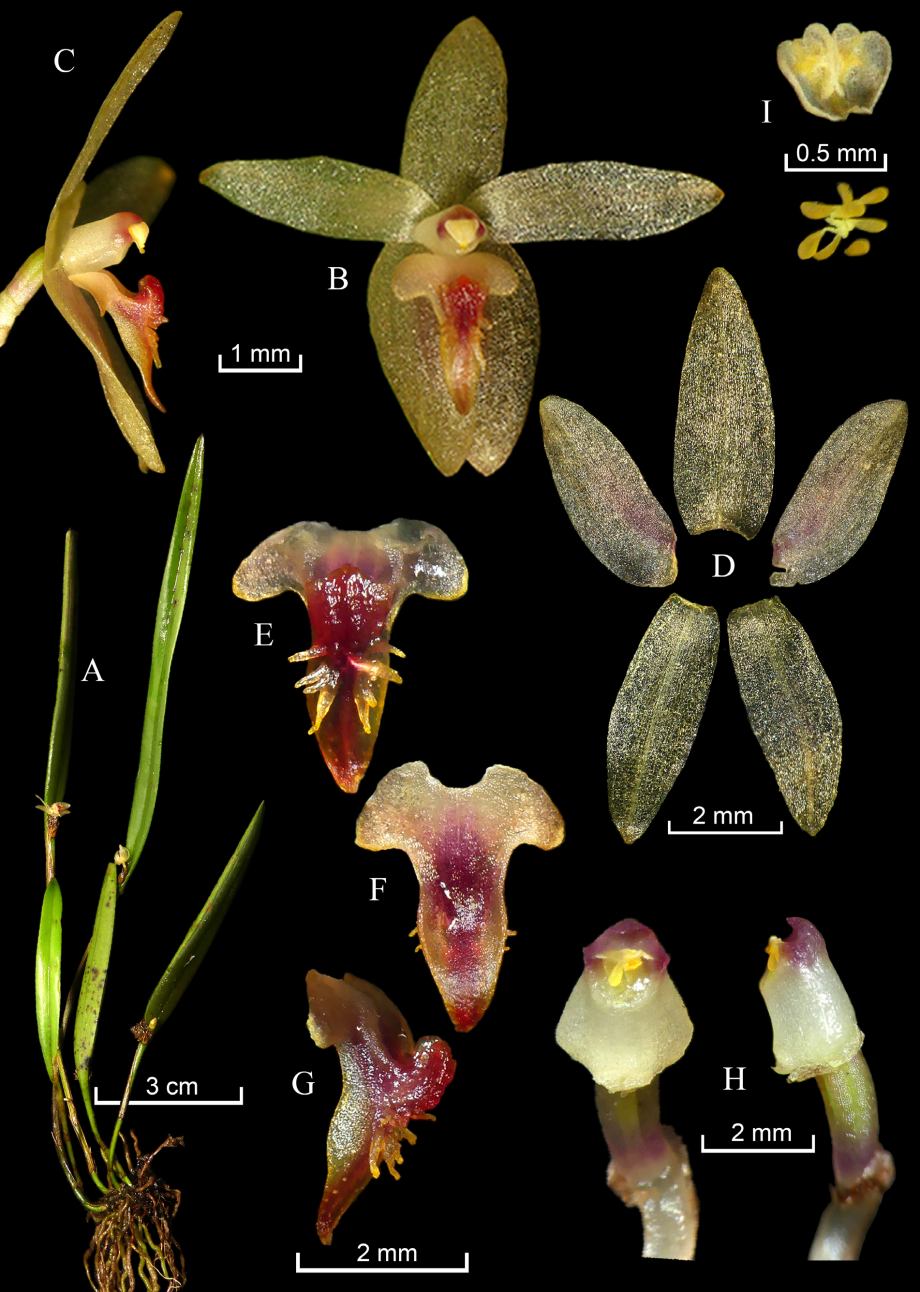
*Octomeria oncidioides*. **A**. Habit; **B**. Flower in frontal and lateral **(C)** views; **D**. Dissected perianth; **E**. Lip adaxial view; **F**. Lip, abaxial view; **G**. Lip, lateral view; **H**. Column ventral (left) and lateral view (right); **I**. Anther, abaxial view (uppermost) and pollinia (lowermost). Photographs by M. M. Jiménez. Plate made by G.A. Iturralde and H.X. Garzón, based on *M. Jiménez 2670*.

*Octomeria oncidioides* (Fig. 6) was previously known only from its type locality in Bolivia, where it was found in premontane Andean-Amazon transitional forest in the north-west of the country. In Ecuador, it was found in the south-eastern provinces of Morona-Santiago and Zamora-Chinchipe, where it is seen growing in similarly transitional forest at similar elevations. These are the first records of this species for Ecuador and though it may seem discontinuous in distribution in relation to the location of its type specimen, it is highly likely to occur across similar habitats along the foothills of the eastern Andes of Perú.

**Figure 7.**
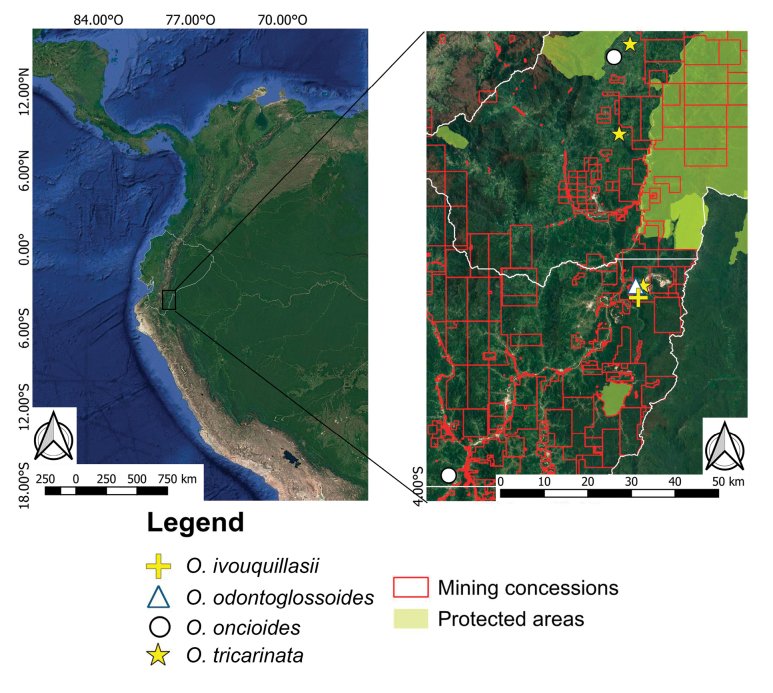
*Octomeria ivouquillasii*, *O. tricarinata* in Ecuador and the other species included in this study. Map elaborated by Gabriel A. Iturralde.

The population found in San Juan Bosco was observed growing on branches of *Alchornea grandis* and *Ficus* sp. in a regenerating secondary forest between 1000 and1200 m elevation. In the second population found near Cumbaratza, *O. oncidioides* was seen growing only on fallen branches.

#### Specimens examined.

Ecuador • Morona-Santiago: San Juan Bosco, 3°5'0286"S, 78°32'21.5"W, 1174 m elev., 3 Feb 2023, *H. Garzón-Suárez 177* (HUTPL 14793!); Ecuador • Zamora-Chinchipe: Near Cumbaratza, 14 Sep 2025, 3°59'30.74"S, 78°53'27.60"W, 1350 m elev., *M.M. Jiménez & M. Jiménez Villalta 2670* (HUTPL 15846!).

##### Note about *Octomeria candidae*

*Octomeria candidae* was described in December 2020 from a population discovered during an orchid diversity survey near El Pangui in the Cordillera del Cóndor of south-eastern Ecuador. The type material was deposited at the Herbario Amazónico del Ecuador under collection number “LV-004”.

In accordance with Art. 9.2 of the International Code of Nomenclature (ICN), the authors hereby correct the citation of the holotype’s collection number. The original publication cited “LV-004” corresponds to a different taxon; the correct collection number for the holotype of *O. candidae* is LV-002. This correction is essential to maintain the integrity of the nomenclatural type. Furthermore, to complement the original description and in compliance with the provisions for valid publication of associated specimens, a paratype is formally designated as follows:

### Octomeria candidae

Taxon classification

Plantae

AsparagalesOrchidaceae

Vélez-Abarca, M.M.Jiménez & Baquero, Lankesteriana 20(3): 347. 2020.

6E069F43-D3C5-5D35-8A21-20D72215B027

#### Type.

Ecuador • Zamora-Chinchipe: Cordillera del Cóndor flank, 890 m elev., 18 Feb 2020, *L. Vélez-Abarca LV-002* (type: ECUAMZ-12211!); ***Paratype***. (here designated) Ecuador • Zamora-Chinchipe: El Pangui, 28 October 2020, *L. Vélez-Abarca LV-066* (paratype: ECUAMZ-12202!).

## Supplementary Material

XML Treatment for Octomeria ivouquillasii

XML Treatment for Octomeria tricarinata

XML Treatment for Octomeria odontoglossoides

XML Treatment for Octomeria oncidioides

XML Treatment for Octomeria candidae

## Additional information

### Artificial Intelligence (AI) use

The authors accept full responsibility for the content of the manuscript, including the disclosure of any use of AI.

No AI tools were used in the preparation of this manuscript.

### Author ORCIDs

L. Vélez-Abarca https://orcid.org/0000-0003-3764-9682

G.A. Iturralde https://orcid.org/0000-0003-2456-0929

H.X. Garzón-Suárez https://orcid.org/0000-0003-4049-1652

D. Gutiérrez del Pozo https://orcid.org/0000-0003-3611-7932

J.R. Kuethe https://orcid.org/0000-0001-5390-9654

L.E. Baquero https://orcid.org/0000-0002-1444-5727

M.M. Jiménez https://orcid.org/0000-0002-9502-5651

### Data availability

All of the data that support the findings of this study are available in the main text.
